# Highly Aligned Bacterial Nanocellulose Films Obtained During Static Biosynthesis in a Reproducible and Straightforward Approach

**DOI:** 10.1002/advs.202201947

**Published:** 2022-07-21

**Authors:** Nerea Murugarren, Soledad Roig‐Sanchez, Irene Antón‐Sales, Nanthilde Malandain, Kai Xu, Eduardo Solano, Juan Sebastian Reparaz, Anna Laromaine

**Affiliations:** ^1^ Institut Ciencia de Materials de Barcelona (ICMAB‐CSIC) Campus UAB Bellaterra 08193 Spain; ^2^ NCD‐SWEET beamline ALBA Synchrotron Light Source Carrer de la Llum 2−26 Cerdanyola del Vallès Barcelona 08290 Spain

**Keywords:** aligned fibers, anisotropy, bacterial nanocellulose, biofabrication, bioinspiration, hydrogels, nanomaterials, sustainability, thermal conductivity

## Abstract

Bacterial nanocellulose (BNC) is usually produced as randomly‐organized highly pure cellulose nanofibers films. Its high water‐holding capacity, porosity, mechanical strength, and biocompatibility make it unique. Ordered structures are found in nature and the properties appearing upon aligning polymers fibers inspire everyone to achieve highly aligned BNC (A‐BNC) films. This work takes advantage of natural bacteria biosynthesis in a reproducible and straightforward approach. Bacteria confined and statically incubated biosynthesized BNC nanofibers in a single direction without entanglement. The obtained film is highly oriented within the total volume confirmed by polarization‐resolved second‐harmonic generation signal and Small Angle X‐ray Scattering. The biosynthesis approach is improved by reusing the bacterial substrates to obtain A‐BNC reproducibly and repeatedly. The suitability of A‐BNC as cell carriers is confirmed by adhering to and growing fibroblasts in the substrate. Finally, the thermal conductivity is evaluated by two independent approaches, i.e., using the well‐known 3*ω*‐method and a recently developed contactless thermoreflectance approach, confirming a thermal conductivity of 1.63 W mK^−1^ in the direction of the aligned fibers versus 0.3 W mK^−1^ perpendicularly. The fivefold increase in thermal conductivity of BNC in the alignment direction forecasts the potential of BNC‐based devices outperforming some other natural polymer and synthetic materials.

## Graphical abstract

Bacteria confined and statically incubated for a few days biosynthesized bacterial nanocellulose (BNC) nanofibers in a single direction without entanglement. The obtained film is highly oriented within the total volume of the film, and it shows a five‐fold increase in thermal conductivity in the parallel direction forecasting the potential of BNC‐based devices outperforming some other natural polymer and synthetic materials.

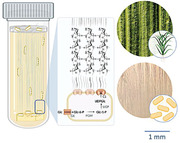

## 1 Introduction

Anisotropic structures are ubiquitous in nature and our tissues, and we find them carrying out particular functions, including mass transport, surface lubrication, cell adhesion, and force generation. For instance, aligned actin fibers confer strong muscle resistance, and highly aligned collagen fibers offer a transparent stromal tissue^[^ ^1^, ^2^ ^]^ or strengthen the skin^[^ ^3^ ^]^ and articular cartilages.^[^ ^4^ ^]^ Additionally, the alignment of fibers usually confers novel and exciting properties to polymers, such as piezoelectricity, conductivity, or light‐harnessing properties.^[^ ^5^, ^6^, ^7^, ^8^, ^9^ ^]^

Cellulose is the most abundant biopolymer in nature,^[^ ^10^ ^]^ and it is commonly obtained from plants, even though several organisms also present the ability to produce it, such as algae,^[^ ^11^ ^]^ fungi, and bacteria.^[^ ^12^ ^]^ The nonpathogenic bacteria *Komagataeibacter xylinus* (*K. xylinus*) extrudes bacterial nanocellulose (BNC) fibers at the liquid–air interface.^[^ ^13^, ^14^ ^]^ BNC is a semicrystalline, hierarchical, and fibrillar network that confers properties such as high water holding capacity, porosity, mechanically solid, and elastic, making the biomaterial highly biocompatible and with bio‐welding properties.^[^ ^14^, ^15^, ^16^, ^17^, ^18^, ^19^, ^20^, ^21^, ^22^ ^]^ Those extraordinary properties placed BNC at the intersection of different applications.

BNC field advances to control the intrinsic properties of cellulose fibrils, such as their arrangement, their interactions with proteins and solvents, or the addition of additives to endow novel properties to the material, among others.^[^ ^23^, ^24^, ^25^ ^]^ Understanding the basic material and the novel composites have endorsed its uses in fields ranging from batteries^[^ ^26^, ^27^, ^28^ ^]^ up to skin grafts,^[^ ^17^, ^18^, ^19^, ^29^, ^30^ ^]^ or even heart replacement pouches.^[^ ^31^, ^32^, ^33^ ^]^

**Figure 1:**
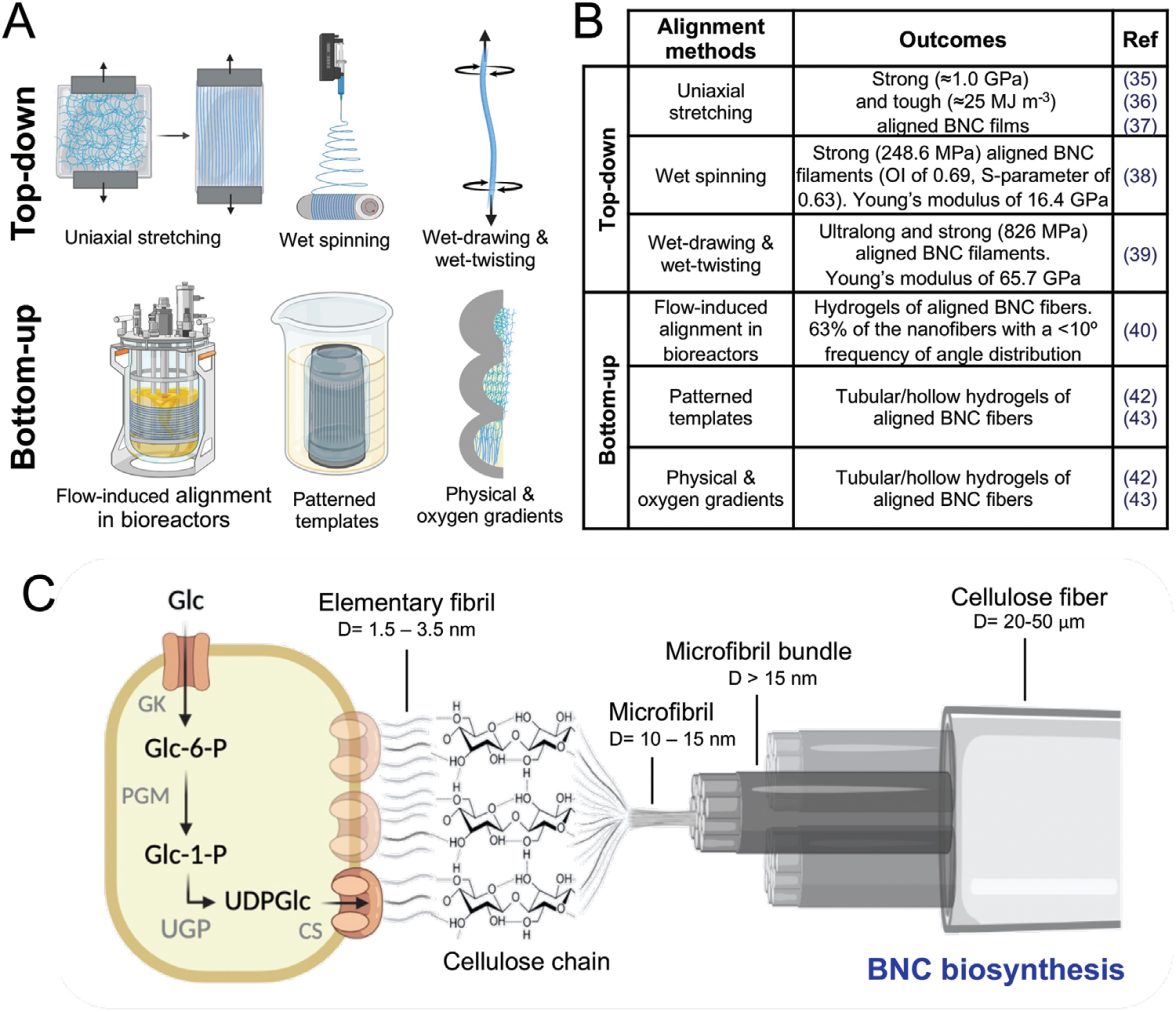
Methodologies to align BNC fibers. A,B) Schematics of the reported methods to align bacterial cellulose fibers such as top‐down approaches: uniaxial stretching,^[^ ^35^, ^36^, ^37^ ^]^ wet spinning,^[^ ^38^ ^]^ and wet‐drawing and wet‐twisting^[^ ^39^ ^]^ to name some and bottom‐up such as flow‐induced alignment in bioreactors,^[^ ^40^ ^]^ patterned templates,^[^ ^42^, ^43^ ^]^ physical and oxygen gradients.^[^ ^42^, ^43^ ^]^ Bottom‐up methodologies benefit from the use of BNC biosynthesis. C) Illustration of the natural BNC biosynthesis and the structural hierarchy of BNC fibers. *K. xylinus*, a cellulose‐producing bacteria strain, synthesizes cellulose from the glucose present in the culture media through the enzymes GK (glucokinase), PGM (phosphoglucomutase), UGP (UDPGlc pyrophosphorylase), and CS (cellulose synthase).^[^ ^43^ ^]^ Cellulose is first produced as elementary fibrils, a cellulose chain of glucose units bound by *β*‐1,4 glycosidic bonds then assembled into microfibrils, which are in turn assembled into microfibrils bundles. The last assembly is the cellulose fiber, which in the standard biosynthesis process in static forms a BNC pellicle at the liquid–air interface. The illustration scales are not representative of reality. D = diameter.

Cellulose and bacterial nanocellulose fibers are promising building blocks for high‐performance biomaterials, especially if we can control their intrinsic mesh structure. BNC fibers are long, flexible, and highly entangled, as obtained; therefore, controlling the alignment of nanofibers is challenging. As reviewed by Li et al.^[^ ^34^ ^]^ different approaches have been presented since bacterial cellulose fibers’ alignment could have interesting properties. Top‐down and bottom‐up approaches have been reported, as illustrated in **Figure** **1**A,B. Top‐down methods, such as an uniaxial stretching of BNC wet hydrogels,^[^ ^35^, ^36^, ^37^ ^]^ the use of wet spinning^[^ ^38^ ^]^ of a BNC solution, and wet‐drawing and wet‐twisting of BNC films^[^ ^39^ ^]^ produced strong, long, and tough aligned BNC films. Yao et al.^[^ ^38^ ^]^ obtained highly aligned BNC, which they evaluated with the orientation parameters^[^ ^34^ ^]^ Orientation Index (OI) and Herman's order parameter (S‐parameter) (Figure 1B), parameters widely accepted to describe anisotropic fibrillar materials.^[^ ^34^ ^]^ However, to keep the alignment structure stable, TEMPO (2,2,6,6‐Tetramethylpiperidinyloxy, a mediator in radical chemistry) oxidation processes, wet spinning of the BNC fibers solutions, stretching and ionic crosslinking of the BNC substrate were required. On the other hand, bottom‐up strategies produced oriented materials by bacteria biosynthesis. Luo et al.^[^ ^40^ ^]^ obtained oriented BNC fibers by forcing the movement of the culture in a single direction in a bioreactor. While an interesting approach, this methodology produced bacteria mutations, decreasing the production yield.^[^ ^40^, ^41^ ^]^ Significantly, Putra et al.^[^ ^42^ ^]^ and Greca et al.^[^ ^43^ ^]^ also benefitted from the bacterial culture and obtained aligned BNC using interesting, although complex, systems to produce and control oxygen and physical gradients in patterned and/or modified surfaces.

Due to the difficulty of maintaining the aligned structures stable,^[^ ^34^, ^41^ ^]^ polymers (PVA or PLGA), ionic crosslinkers^[^ ^38^ ^]^ or other additives are often required to improve and stabilize the aligned BNC films. For example, Rahman et al.^[^ ^36^ ^]^ added soy protein to uniaxial‐stretched BNC films to enhance the mechanical properties by 2.75 and threefold in tensile strength and tensile modulus, respectively.

Even though there are some examples of materials containing aligned BNC fibers, yet the methods to produce them are not straightforward. We designed a methodology to obtain aligned BNC films in static conditions and avoid complex equipment or chemical additives. For that, we observed the BNC synthesis. *K. xylinus* produces cellulose, as described in Figure 1C, involving a chain of biochemical reactions. Cellulose fibers are extruded linearly through the bacteria cell wall's pores,^[^ ^6^, ^14^, ^44^, ^45^ ^]^ as recorded by R. Malcolm Brown, JR. laboratory.^[^ ^45^ ^]^ Cellulose ensembles in hierarchical structures from elementary fibrils to BNC fibers^[^ ^21^, ^22^ ^]^ (terminology used in the text), and forms a final BNC pellicle at the liquid–air interface in static biosynthesis. Motivated by this biosynthesis process, we investigated whether the restriction of the bacteria's movement could retain the intrinsic parallel orientation of individual cellulose fibers.^[^ ^44^ ^]^ Here, we report a novel, reproducible, and easy biofabrication method to obtain aligned BNC (A‐BNC) hydrogels with high control, avoiding complex equipment and additives. The morphology, optical activity, and how the anisotropy of this material impacts its hydrophilicity, biocompatibility, and thermal conductivity of A‐BNC films are evaluated.

## 2 Results and Discussion

### 2.1 Aligned Bacterial Cellulose Biosynthesis

**Figure 2:**
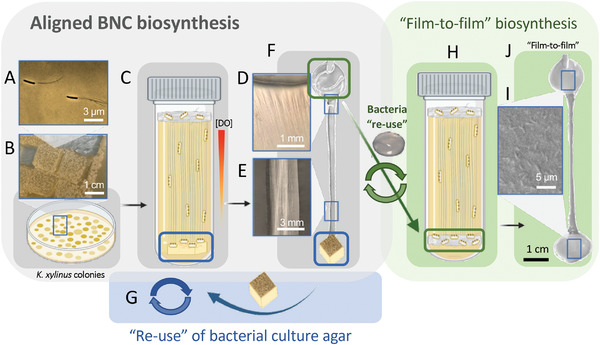
Methodology to obtain aligned BNC (A‐BNC) and the reuse of the bacterial substrates. A) Screenshot of the extrusion of BNC fibrils by *K. xylinus* strain's individual pores when immersed in HS liquid culture media with no restriction of movement. B) *K. xylinus* colonies grow confluent on the surface of HS solid media (agar). C) Square section of agar with confluent bacterial colonies (marked in blue) is placed at the bottom of the culture tube filled with HS liquid culture media, restricting the bacteria movement. Due to the oxygen availability, bacteria move toward the liquid–air interface to reach higher DO concentrations, leaving aligned BNC (A‐BNC) fibers behind. Hence, A‐BNC is synthesized with directionality toward the liquid–air interface of the culture, forming BNC. D,E) Optical images of the macrostructure of A‐BNC within the length of a A‐BNC and BNC film. F) Reuse of bacterial substrates: agar containing bacterial colonies (marked as blue) and the produced BNC film (marked as green) are reused to grow aligned BNC. G) Reusing the agar square piece, which still maintains bacterial confluence, at the bottom of the vessel of a new culture tube allows obtaining A‐BNC and BNC repeatedly for at least 7 cycles. H) Reusing the produced and not cleaned BNC film (marked in green in F) allowed the “Film‐to‐film” biosynthesis method. I) Scanning electron microscopy (SEM) image of an uncleaned BNC film containing bacteria. J) Reusing uncleaned BNC films in a new culture tube allowed obtaining “Film‐to‐film,” which consist in 2 BNC films bound at the extremes of an A‐BNC film. This process can be repeated multiple times and avoiding bacteria and time waste.

*K. xylinus* is an aerobic bacteria strain^[^ ^6^, ^44^ ^]^ and typically produces BNC films which at harvested at the liquid–air interface in the culture. Bacteria in solution can move without any restriction of movement Figures 1C and **2**A and drives the production of the water‐insoluble^[^ ^14^ ^]^ cellulose nanofibrils toward a higher oxygen content.^[^ ^42^, ^43^ ^]^ In our approach, we first cultured confluent *K. xylinus* colonies on an agar surface containing nutrients for 15 days at 30 °C (Figure 2B). Then, we cut square pieces of the agar with the colonies and place those agar squares with the bacterial colonies facing up at the bottom of a vessel, previously filled with fresh bacteria‐free liquid media. Bacteria anchored at the agar started to produce cellulose at 30 °C in static conditions (Figure 2C; and Figure S1, Supporting Information). As previously reported, we obtained the typical hydrogel pellicle of BNC at the liquid‐air interface after 5 days of culture^[^ ^14^, ^44^ ^]^ (Figure 2C). The appearance of this film indicated that some bacteria reached the surface from the vessel's bottom and were able to produce cellulosic fibers at the liquid–air interface. As a result of the migration from the vessel's bottom to the surface, a translucent film of aligned BNC fibers is formed in the liquid spanning the whole length of the culture vessel after a biosynthesis cycle, as shown in Figure 2D,F. This methodology was repeated *n* ≥ 200, obtaining freestanding A‐BNC and BNC films consistently (Figure S2A, Supporting Information). From each culture, we isolated the aligned cellulose (A‐BNC), which was bound to the agar substrate from the bottom of the vessel and to the cellulose hydrogel pellicles obtained at the top of the vessel (BNC) (Figure 2D). The restriction of the bacteria movement allowed to produce fibers without the entanglement in a standard bacterial liquid culture. Hence, we obtained BNC films of different morphologies (aligned and nonaligned) in a straightforward step.

We cleaned the films from bacteria detritus and culture media using the standard treatment described previously.^[^ ^46^ ^]^ We obtained semitransparent hydrogels of BNC of the vessel's shape, in our case disks of Ø1.6 cm,^[^ ^2^ ^]^ and aligned bacterial nanocellulose (A‐BNC) of 7–8 cm in length. A‐BNC and BNC films were dried at 60 °C for a minimum of 12 h. After drying, A‐BNC maintained the aligned structure and length observed in the wet state. A‐BNC films were easily manipulable and flexible, with a thickness of ≈10 µm after drying. Optical images of the A‐BNC clearly show the alignment of the fibers (Figure 2C,D); conversely, the random BNC does not show any orientation of its structure.

Interestingly, A‐BNC did not disassemble upon different wetting‐drying cycles and the whole cleaning process, indicating a stable cellulose fibers interaction conferred by hydrogen bonds between hydroxyl groups.^[^ ^10^ ^]^ This is a key feature as reported aligned films usually require additives and crosslinkers to maintain the structure.^[^ ^34^, ^38^ ^]^

Many parameters control the BNC production, and they have been evaluated elsewhere.^[^ ^14^, ^43^, ^46^ ^]^ However, the media's dissolved oxygen (DO) concentration is considered the main driver (Figure 2C). We quantified the DO concentration present in the solution at different time points using the Leuco‐indigo carmine reaction^[^ ^47^, ^48^ ^]^ (Figure S3, Supporting Information) in our system. Leuco‐indigo carmine reagent oxidizes under the presence of DO to keto‐indigo carmine, which in turn causes a color change in the solution from yellow to red upon oxidation (Figure S3A, Supporting Information). A color chart allows to qualitatively estimate the DO concentration (Figure S3B, Supporting Information). As shown in Figure S3C (Supporting Information), the initial DO concentration at the top, and bottom part of a nonoxygen permeable tube was ≈0.06 ppm. After 7 days of culture, the DO concentration at the top was maintained at 0.06 ppm while, at the bottom, it decreased to 0.03 ppm. We hypothesize that the deployment of oxygen at the bottom of the vessel over time promotes the movement of the bacteria to the surface toward higher DO content. Thus, bacteria produce water‐insoluble cellulose fibers from the bottom of the vessel and use them as “climbing ropes” (Figure 2C) to reach the high oxygen‐content surface where they continue producing its exoproduct with no movement restriction, allowing us to obtain BNC as a random mesh. Remarkably, we efficiently obtain A‐BNC and BNC from the same biosynthesis process by restricting the initial movement of bacteria and its entanglement.

To further exploit the efficiency and yield of our new method, we investigated the reuse of the agar containing bacterial colonies and the produced and uncleaned BNC films (marked in blue and green in Figure 2F, respectively). After an A‐BNC and BNC film culture, the agar substrates did not lose the confluence of bacterial colonies, which confirmed its suitability for our methodology based in the restriction of the bacteria movement. In Figure S4 (Supporting Information), bacterial colonies can be observed at the extreme of an A‐BNC and BNC film, which was pulled from the agar substrate. This piece of agar was carefully released from the produced cellulosic film and confined again at the bottom of a new culture tube previously filled with fresh liquid culture media (free of bacteria) (Figure 2G). We were able to reuse the same agar substrates with confluent bacterial colonies (Figure 2G) for at least 7 cycles, obtaining A‐BNC and BNC films in only 3 days. After that, the bacteria decreased the speed of production. *K. xylinus* exhibits the growth dynamics of a common bacteria, decreasing its activity and efficiency to produce BNC over time since it enters the death phase, where there is less metabolic activity.^[^ ^41^, ^49^ ^]^ We also explored the reuse of the uncleaned BNC film produced at the liquid–air interface, which we named “Film‐to‐film” synthesis (Figure 2H). By scanning electron microscopy (SEM) we detected the presence of bacteria in uncleaned BNC films (Figure 2I). As shown in Figure 2H, uncleaned BNC films (marked in green) containing live and active bacteria allow producing A‐BNC films resembling agar substrates. BNC films could easily be detached from A‐BNC by pulling off the film with Teflon tweezers and cutting with sterilized scissors. When placed at the bottom of a vessel, the bacteria immediately produce cellulose fibers. This results in films containing 2 circular BNC pellicles bound at the extremes of an A‐BNC film (a “Film‐to‐film”, Figure 2J), which allows the production (2^n^) of A‐BNC films. This process could be used repeated for at least 7 cycles, obtaining freestanding “Film‐to‐film” consistently (Figure S2B, Supporting Information). The re‐use of agar and uncleaned BNC film substrates in the A‐BNC production allowed to increase exponentially the number of films obtained. We hypothesize that the already‐used bacteria are acclimatized to the liquid culture, reducing the lag phase of a bacteria culture from the initial 5 days of the first culture to only 3 days, speeding the biosynthesis process.^[^ ^41^, ^49^ ^]^

To increase the dimensions of the A‐BNC produced, we evaluate the culture time, vessel dimensions, and bacteria amount. Culturing the bacteria from 3 to 15 days did not increase the width or length of A‐BNC, although it increased the thickness of the BNC film. On the other hand, longer A‐BNC films could be obtained using longer culture tubes. Using tubes of 10 and 30 cm allowed obtaining A‐BNC 7–8 cm up to 17–20 cm, respectively; however, the width of the A‐BNC was not increased (3–5 mm). Figure S5 shows images of the methodology to obtain A‐BNC films in large vessels (30 cm). Finally, instead of using the typical streak plate (or zig–zag) method to obtain the initial agar substrate with bacterial colonies,^[^ ^50^ ^]^ we grew confluent agar Petri dishes to maximize the number of bacteria per surface to obtain high‐density A‐BNC fiber films. This strategy allowed us to obtain A‐BNC films with a thickness of ≈200 µm in wet.

### 2.2 A‐BNC Characterization

We extensively characterized A‐BNC and BNC (considered a control) to evidence the change of morphology and evaluated the properties arising upon the alignment of the fibers.

**Figure 3:**
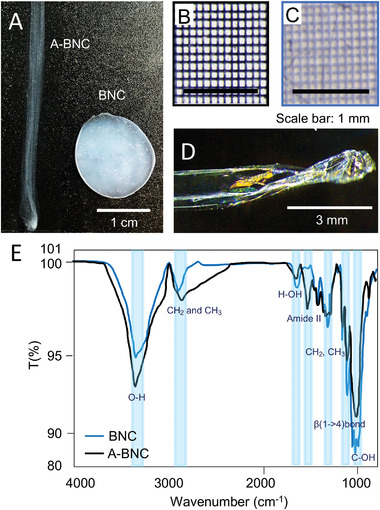
Transparency and chemistry of A‐BNC. A) Pictures of A‐BNC and BNC, respectively. B,C) Pictures of A‐BNC and BNC on top of a micrometer‐mesh, indicating a hazier BNC than A‐BNC. D) Dry A‐BNC shows iridescence effects under the incidence of white light. E) FTIR reveals that A‐BNC and BNC have similar chemical compositions. However, the A‐BNC spectra show more intense transmittance peaks in the OH and CH region.

After drying, A‐BNC is more transparent than BNC, qualitatively appreciated by the naked eye (**Figure** **3**A). Figure 3B,C show pictures taken with a micrometer‐mesh pattern under the A‐BNC and BNC films, respectively, and top illumination. The scattering of light of BNC produced a haze and whitish color to the material that was not seen in the A‐BNC. Moreover, under the incidence of white light, the A‐BNC films showed iridescence, indicating higher transparency and structuration of the material (Figure 3D).

As seen in Figure 3E, FTIR for both samples (BNC (blue) and A‐BNC (black)) contained the cellulose characteristics peaks: hydroxyl groups are seen as a strong peak at 3000–3700 cm^−1^, CH groups at 2800–3100 cm^−1^, and the internal deformation frequencies of CH_2_ and CH_3_ groups appeared in the region of 1340–1480 cm^−1^. However, the A‐BNC spectra show more intense transmittance peaks in the OH and CH region, characteristic of cellulose, even though A‐BNC films were thinner than the BNC.

**Figure 4:**
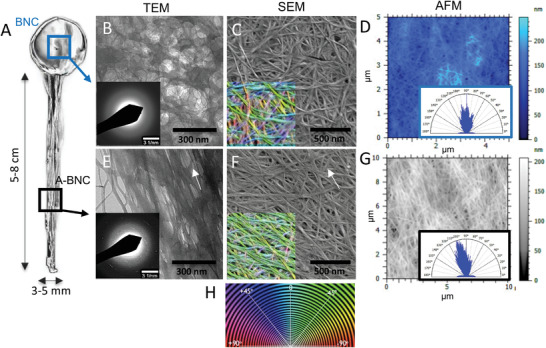
Morphology and structure analysis. A) Picture of the as‐produced nanocellulose films containing BNC and A‐BNC. The top B,C,D) and bottom E,F,G) rows contain the images obtained by TEM, SEM, and AFM of BNC and A‐BNC. B,E) TEM images and selected area electron diffraction (SAED) patterns of BNC and A‐BNC samples. C,F) SEM micrographs of BNC and A‐BNC samples (Insets: color analysis from the *OrientationJ* plugin for ImageJ). D,G) AFM images of BNC and A‐BNC samples (Insets: isotropy distribution computed using MountainView8).

To analyze the BNC film at the nanoscale (**Figure** **4**), films were dried, which collapsed the structure and made the alignment observed at the macroscale more difficult. Even though a higher degree of organization was observed in the A‐BNC than in BNC. Figure 4B,E shows the transmission electron microscopy (TEM) images and selected area electron diffraction (SAED) patterns of BNC (Figure 4B) and A‐BNC (Figure 4E). Although BNC is semicrystalline (60–80%),^[^ ^44^ ^]^ the thickness of the film, compactness, and orientation of the fibers make it challenging to analyze the crystallinity of the BNC pellicle by TEM.^[^ ^51^ ^]^ SAED analysis in A‐BNC samples allowed visualizing the cellulose crystallinity lattice planes (indexed in Figure S6A, Supporting Information). We considered that this increased discretization of the lattice planes intensity (from diffuse bands to localized bright spots, Figure S6, Supporting Information) was due to the increased order orientation and different textures of A‐BNC. Scanning electron microscopy (SEM) and atomic force microscopy (AFM) images show a similar compact structure for the BNC (Figure 4C,D) and A‐BNC (Figure 4F,G) films; however, we could see the alignment of fibers in A‐BNC. Diameter measurements of the nanofibers (2–4 nm) and microfibrils bundles (15–20 nm) from both A‐BNC and BNC revealed that the modification in the culture to obtain aligned BNC did not impact the single fiber structure. Using the color analysis from the *OrientationJ* plugin for ImageJ, we computed the local orientation as a color‐survey HSB image, where hue represents the fiber orientation, and saturation represents orientation coherency. These images (insets of Figure 4C,F) indicated the preferential fiber orientation in degrees. We can see that A‐BNC had a predominance of colors in the green (Figure 4F) region, indicating a predominant direction (pointed by white arrows for a more precise visualization) not appreciable for BNC samples (Figure 4C), where a variety of colors represent the different orientations within the image. The mean height of AFM images analyzed by MountainView8 was 28.6 nm for BNC and 21.1 nm for A‐BNC, indicating a flatter surface. Additionally, we computed the isotropy of the pictures in Figure 4D,G, showing higher values for BNC, 31%, in contrast with A‐BNC, 19%, confirming the alignment. Interferometry assisted us in visualizing the alignment at a micron scale, obtaining volumetric images of the aligned cellulose BNC bundles (Figure S7A, Supporting Information), in contrast with the nonaligned BNC (Figure S7B, Supporting Information), where nonbundle ordering is appreciated.

**Figure 5:**
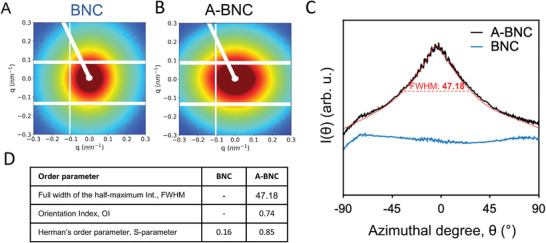
Nanoscale density study with SAXS. A,B) Small angle X‐ray scattering (SAXS) scattering signal of BNC and A‐BNC. C) Integration of the spectra as intensity versus azimuthal degree plot, from −90° to 90°, whereas BNC gave no peak. D) Table with the quantitative orientation parameters orientation index (OI), full width at the half‐maximum intensity (FWHM), and Herman's order parameter (S‐parameter) values for BNC and A‐BNC.

To evaluate the alignment in the 3D volume of the material, we used interferometry, small angle x‐ray scattering (SAXS), and polarization‐resolved second‐harmonic generation signal (P‐SHG). The raw 2D scattering SAXS patterns of A‐BNC and BNC fibers showed a different scattering pattern shape, being circular for randomly distributed fibers of the BNC (**Figure** **5**A) and distorted (elliptical) for preferential orientated fibers of the A‐BNC sample (Figure 5B), indicating a high degree of alignment. The spectra were integrated to quantitatively evaluate differences in the orientation degree, as shown in Figure 5C, as of intensity versus azimuthal degreeplot from −90° to 90°. A‐BNC exhibited an intensity peak centered around 0°, whereas BNC gave no peak. From the widely accepted orientation quantitative parameters orientation index (OI), full width at the half‐maximum intensity (FWHM), and S‐parameter^[^ ^34^, ^38^ ^]^ obtained from the spectra where FWHM stands for full width at the half‐maximum intensity and the smaller this value is, the highest is the orientation. The OI is obtained from FWHM, ranging from 0 for randomly oriented structures to 1 for perfectly aligned fibers. Herman's order parameter, also called S‐parameter, can be calculated from the intensity and azimuthal degrees values, and it ranges between 0 and 1, being 1 a perfect oriented.^[^ ^31^, ^34^ ^]^ Thus, as shown in Figure 5D, FWHM, OI, and S‐parameter were computed, obtaining 47.18°, 0.74, and 0.85 for A‐BNC, respectively. For BNC, S‐parameter resulted to be much smaller than A‐BNC, 0.16. FWHM and OI could not be determined due to the no peak presence in the spectra, thus lacking internal orientation. The numerical results obtained corroborated the qualitative information extracted from the 2D images. The orientation values OI and S‐parameter (0.74 and 0.85, respectively) were higher than previously reported values of aligned BNC fibers obtained by wet spinning, with OI of 0.69 and an S‐parameter of 0.63.^[^ ^31^ ^]^

**Figure 6:**
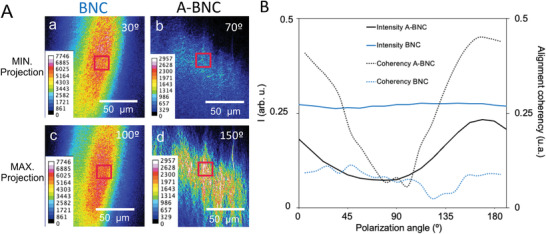
Polarization‐resolved second harmonic generation (PSHG) microscopy. Stacks of 18 images (frame/10° light polarization) were analyzed with ImageJ using a heat map and Z projections. A) Maximum and minimum SHG intensity projection of 150 × 150 µm^2^ for BNC and A‐BNC, along with the intensity color map. B) Plot of the SHG intensity for each angle polarization, along with their alignment coherency, obtained with the *OrientationJ* plugin for ImageJ.

Secondary harmonic generation (SHG) is a nonlinear optical process used in biomedicine for imaging SHG active biological structures such as collagen nanofibers, rather than bulk.^[^ ^43^, ^52^, ^54^ ^]^ Polarization‐resolved SHG microscopy (PSHG) is a powerful tool that exploits the dependence of SHG signals on the polarization degree of the excitation beam.^[^ ^52^ ^]^ The SHG emission depends on the structuration of the imaged material and PSH includes an extra dimension that is used to probe the molecular organization. Here, PSHG was used to assess the bacterial nanocellulose intrinsic organization. Frames of the linear polarization of the incident beam from 0° to 180° in steps of 10° using a *λ*/2 plate were recorded for the whole volume of the samples. **Figure** **6**A shows the changes of light intensity in A‐BNC from the minimal (70°) to the maximum projection (150°), whereas, in BNC, all projections remained unchanged, indicating no alignment. These features were observed for the whole thickness of the samples, ensuring a volumetric alignment in A‐BNC. We evaluated the alignment coherency for each frame by the *OrientationJ* plugin for ImageJ (Figure 6B). Random BNC displays a high but invariable signal intensity (blue line) and a low alignment coherency (dotted blue line). Conversely, A‐BNC alignment coherency (dotted black line) is even higher than the signal intensity (black line) at the maximum projection and lower in the minimum projection, confirming the alignment of the material.

### 2.3 Optical Properties

**Figure 7:**
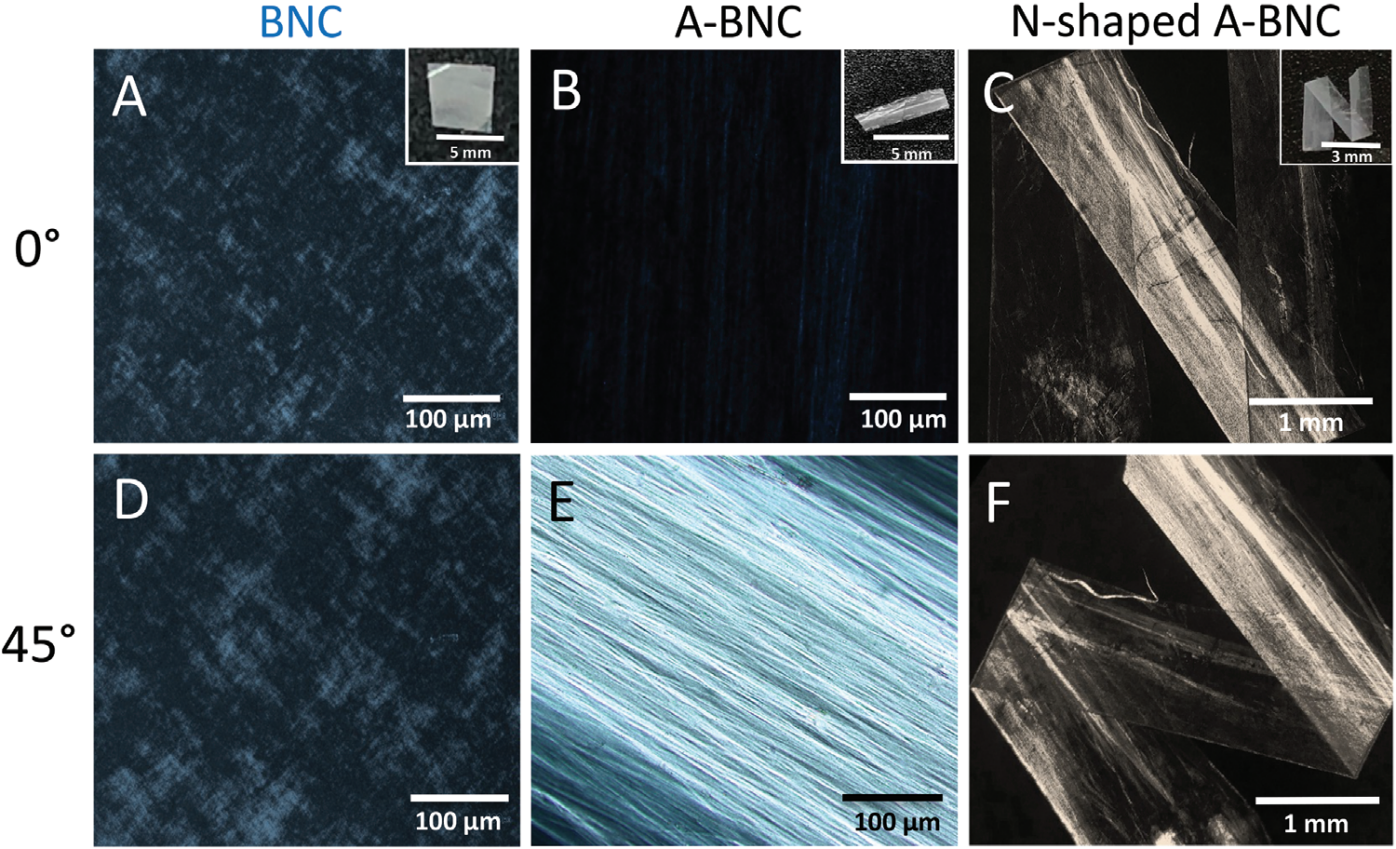
The behavior of A‐BNC and BNC under polarized light. Visualization under a polarized light microscope at 0° and 45° (incident polarized light beam vs sample orientation) of A,D) BNC samples, B,E) A‐BNC samples and C,F) N‐shaped A‐BNC (Insets of whole films in (A)–(C)). Samples are circularly rotated within the same plane, perpendicularly to the incident polarized light beam.

As we have presented, A‐BNC films exhibit iridescence and strong light polarization properties. The use of polarized light is a well‐known methodology to assess a material's anisotropy.^[^ ^40^, ^42^ ^]^ As shown in **Figure** **7**, a dark‐light patterns can be obtained when a sample is circularly rotated 45°. The minimum light intensity is obtained when the fibers are placed at 0° from the incident polarized light, whereas the maximum light intensity of the fibers appear at 45°. BNC films did not exhibit light polarization at 0° and 45° (Figure 7A,D), confirming the random distribution of the fibers. On the other hand, A‐BNC films gave dark‐light patterns of light when disposed at 0° and 45° from the incident polarized light beam, respectively (Figure 7B,E), confirming the anisotropy of the material. Moreover, A‐BNC is a flexible material and can be folded into any desirable shape, such as an “N”. As seen in Figure 7C,F, N‐shaped films of A‐BNC created a pattern of light polarization at different positions.

### 2.4 Hydrophilicity and Cell Substrates Evaluation

**Figure 8:**
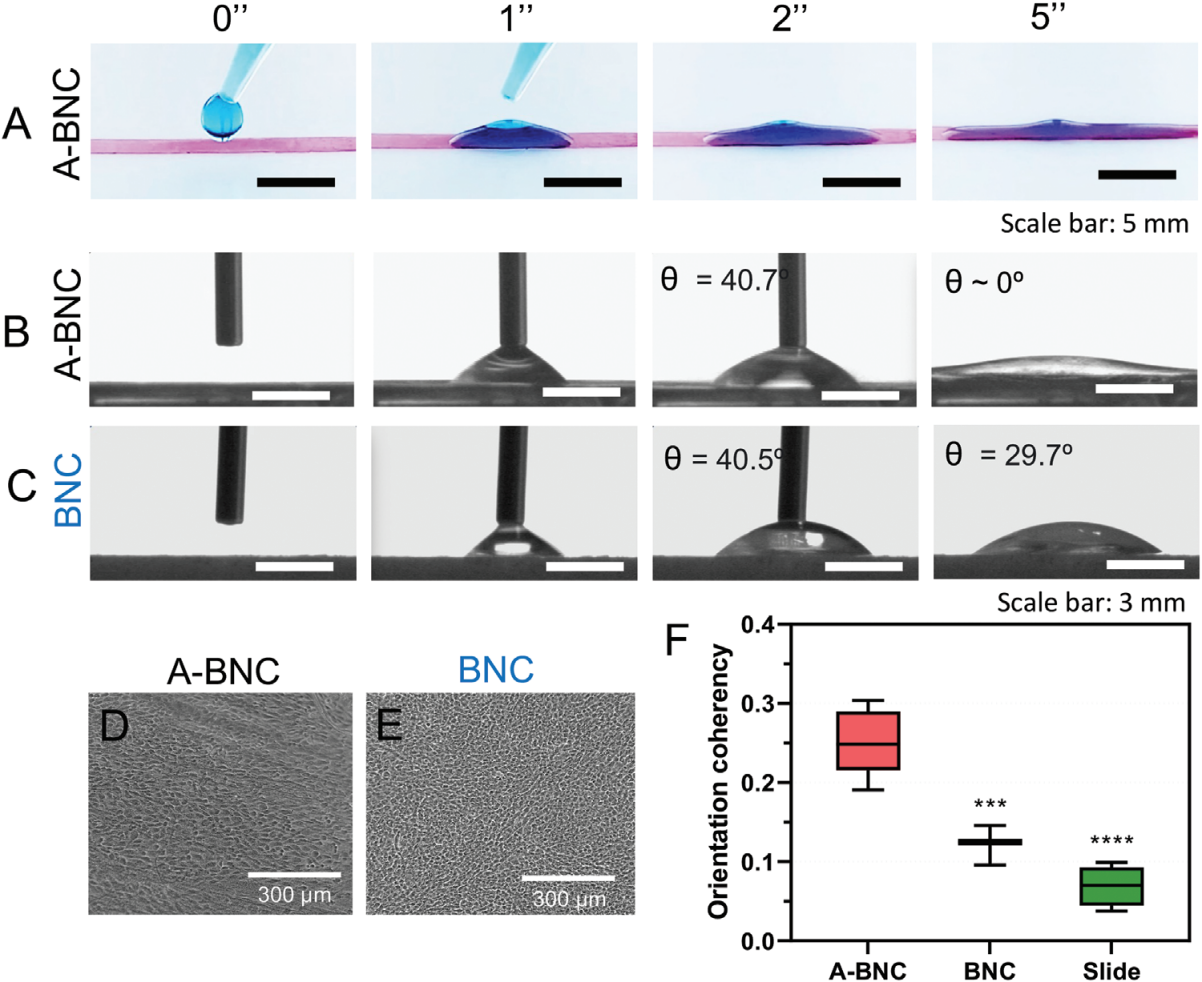
Hydrophilicity and cell substrates evaluation of A‐BNC and BNC. A) Pictures of an A‐BNC film's wetting with a colored water drop. B,C) Measurement of the apparent contact angle (ACA) of A‐BNC and BNC films, respectively. D,E) Optical images of confluent cell cultures at day 7 on A‐BNC and BNC, respectively. F) Orientation coherency plot of A‐BNC, BNC, and the slide (control), computed with the *OrientationJ* plugin of ImageJ. *** = *P* ≤ 0.001, **** = *P* ≤ 0.0001.

Most of the electrospun polymers are relatively hydrophobic, which is unfavorable from the point of view of tissue engineering. For instance, aliphatic polyesters such as Poly(*L*‐lactide) (PLLA) or Polycaprolactone (PCL) show contact angles in the range of 116°–135°, while tissue engineering requirements ought to be below 100°.^[^ ^55^, ^56^ ^]^ Webb et al. and Niemczyk et al. studied the highest level of fibroblasts cell attachment at hydrophilic surfaces,^[^ ^57^, ^58^ ^]^ and the best results were observed for surfaces with contact angles in the range of 20°–40°. As cellulose, and specifically, bacterial nanocellulose drives many applications in medicine^[^ ^59^, ^60^ ^]^ for their biocompatibility and hydrophilic character, we wanted to analyze the impact of the fiber alignment in the hydrophilic nature of bacterial cellulose. Some reports already suggest the importance of structuration of cellulose on the growth of specific cell lines.^[^ ^33^, ^61^ ^]^ We deposited a blue‐colored drop of water over A‐BNC film, and we could see a faster horizontally spreading of the liquid than BNC, indicating a different structure (**Figure** **8**A). To quantify the hydrophilicity of both A‐BNC and BNC, we performed “apparent contact angle” (ACA) measurements. After 5 s of depositing a water drop over the surfaces, we observed an ACA of 0° for A‐BNC (Figure 8B), whereas it remained 29.7° for BNC after 5 s (Figure 8C). These results indicated that the alignment of fibers seemed to increase the hydrophilicity of the films, which also matches with the lower roughness of A‐BNC in AFM and the increased density of OH groups by FTIR.

This change of hydrophilicity was further explored in vitro in cell cultures. A human dermal fibroblast (hDF) cell line (1BR.3.G) was cultured on A‐BNC and BNC films. The cell attachment and growth directionality were assessed visually and with orientation coherency measurements with the *OrientationJ* plugin of ImageJ. As seen in Figure S8 (Supporting Information), cells attached and proliferated on A‐BNC, BNC, and control (glass slide) until reaching a 100% confluence at day 7 (Figure S8, Supporting Information; and Figure 8D,E). As seen in Figure 8F, hDF cells cultured on aligned cellulose fibers tend to be more elongated (orientation coherency of 0.25) than on random fiber distributions (BNC film) (orientation coherency of 0.12) and on the slide (orientation coherency of 0.8) The increase of hDF directionality indicated that cells perceived at some extend the alignment of the cellulosic fibers.

### 2.5 A‐BNC Thermal Conductivity

**Figure 9:**
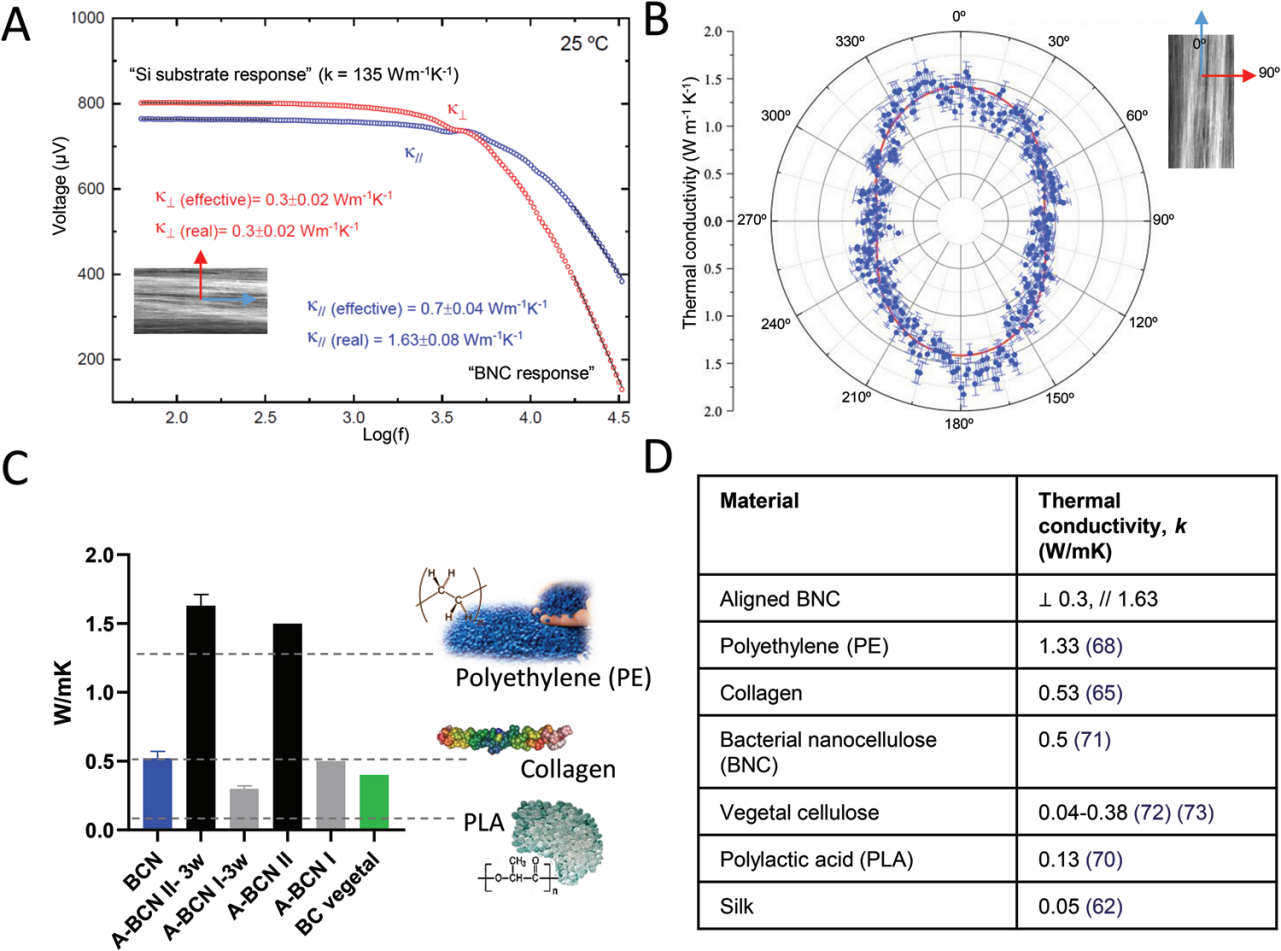
Thermal conductivity study of A‐BNC. A) 3*ω* measurements of A‐BNC at the parallel and perpendicular directions were evaluated, indicating a maximum five‐fold increase of the thermal conductivity in the parallel direction of A‐BNC fibers. B) Thermal conductivity of A‐BNC measured by anisotropic thermoreflectance thermometry, showing similar results as shown in (A). C) Comparison of the obtained measurements to the standard collagen,^[^ ^65^ ^]^ PLA,^[^ ^70^ ^]^ or PE^[^ ^68^ ^]^ used in implants. D) Table of the reported thermal conductivity of polymeric materials.^[^ ^62^, ^65^, ^68^, ^70^, ^71^, ^72^, ^73^ ^]^

The advent of more sophisticated implants combining novel biomaterials, polymers, electrical components, among others, urges precise thermal management.^[^ ^62^ ^]^ Amorphous materials and good thermal insulators tend to be abundant in biologically derived materials; to enhance their thermal conductivity, self‐assembled biomaterials with a higher degree of crystallinity and higher anisotropy are desirable.^[^ ^63^ ^]^ BNC has already shown promising results as a scaffold in regenerative medicine,^[^ ^17^, ^18^, ^19^, ^64^ ^]^ we studied the influence of the geometrical order on the thermal conductivity of BNC fibers. In particular, we show that it is possible to enhance the thermal conductivity by directional alignment of the fibers, i.e., in the direction parallel to the alignment direction. We used two independent approaches to investigate the thermal conductivity of A‐BNC, using the well‐known 3*ω*‐method,^[^ ^65^ ^]^ and a recently developed contactless thermoreflectance frequency‐domain method developed by Pérez et al.,^[^ ^66^ ^]^ named anisotropic thermoreflectance thermometry (ATT). The latter methodology is particularly suitable for studying thermally anisotropic materials (e.g., aligned BNC), since it delivers the angular distribution of the thermal conductivity perpendicular to the sample's surface. In all cases, the BNC fibers were deposited on silicon substrates, and the thickness of the BNC samples was ≈10 µm. **Figure** **9**A displays the 3*ω* measurements in perpendicular (red) and parallel (blue) to the alignment direction. Whereas the lower frequency range is dominated by the thermal response of the Si substrate, the higher frequency range contains information on the thermal properties of the fibers. The thermal conductivity is estimated using the “slope method” (*κ* *α* [∂*V*_3*ω*_/∂ log( *f* )]^−1^) in the higher frequency range. The lower frequency range is dominated by a thermal signal from the substrate, and it is used as calibration for each measurement (*κ*_Si_ = 150 W m^−1^ K^−1^). In the parallel direction, A‐BNC shows a more significant thermal conductivity at 1.63 ± 0.15 W m^−1^ K^−1^, whereas in the perpendicular direction, the thermal conductivity is 0.3 ± 0.02 W m^−1^ K^−1^, slightly lower than what we previously obtained for BNC, 0.5 ± 0.05 W m^−1^ K^−1^.^[^ ^7^ ^]^ The alignment of the fibers led to a fivefold increase of the thermal conductivity of BNC in the parallel direction (Figure 9A). Similar results were obtained with ATT, where thermal conductivity was measured almost continuously with 1° angular steps from the alignment direction (Figure 9B). Those results indicate that the heat transport is more efficient in the parallel direction rather than the perpendicular. The main limitation in the perpendicular direction is the dominant thermal interface resistance between fibers since these are probably interacting. This increase in heat transport in aligned polymers has been already reported, for instance for polyethylene.^[^ ^67^ ^]^ The highest value for A‐BNC, at its parallel direction, (1.42 ± 0.15 W m^−1^ K^−1^), is similar to the value obtained using the 3*ω*‐method (1.63 ± 0.15 W m^−1^ K^−1^), This number is on the same order as polyethylene (PE) (1.33 W m^−1^ K^−1^),^[^ ^68^ ^]^ and larger than collagen (0.53 W m^−1^ K^−1^),^[^ ^65^ ^]^ which are materials commonly used in implants. Therefore, the geometrical alignment of the fibers offers the possibility to control at different planes the thermal conductivity just by tailoring the anisotropy of the biomaterial.^[^ ^69^ ^]^ Interestingly, the perpendicular component of the thermal conductivity from the ATT scans (0.87 ± 0.09 W m^−1^ K^−1^) resulted twice as large as compared to the value obtained using the 3*ω*‐method (0.3 ± 0.02 W m^−1^ K^−1^). The origin of these different values in the perpendicular direction possibly arises from different thermal resistances between parallel fibers. From those results, we foresee the potential of natural‐based materials, which may outperform polymer and synthetic materials in some properties.

## 3 Conclusions

Inspired by the bacterial cellulose biosynthesis, we presented a facile and reproducible method to obtain films of aligned cellulose fibers (A‐BNC) and nonaligned cellulose (BNC) fibers in a single step. Bacteria confined at the bottom of a vessel and statically incubated for a few days biosynthesized BNC nanofibers in a single direction toward the liquid–air interface without entanglement. Cellulose fibers strongly interact, creating an aligned film which alignment is maintained upon different dry‐wetting cycles without the use of any additionally additive. The reuse of bacterial substrates for at least 7 cycles, allowed to speed and increase the production of A‐BNC. Optical, chemical, and physical techniques evaluated confirmed the alignment of the BNC fibers superficially and in the whole volume of the film. Mechanical properties were not reported, to the fragility and low thickness of A‐BNC. However, to facilitate the comparison with reported materials, future work includes the in‐depth mechanical evaluation of A‐BNC films. A‐BNC demonstrated to be suitable as a cell carrier substrate, as fibroblasts adhered and grew. The evaluation of the thermal conductivity of A‐BNC using two independent approaches confirmed a fivefold increase of the thermal conductivity in the parallel direction to the alignment axis, 1.63 ± 0.15 W m^−1^ K^−1^, whereas, in the perpendicular direction, the thermal conductivity is 0.3 ± 0.02 W m^−1^ K^−1^. The properties risen by the alignment encompasses the properties already exploited for celluloses as their easy functionalization, biocompatibility, and purity, which bring cellulose to be used in an even more significant number of fields, and even join the biomaterials’ group of our future and active implants.

## 4 Experimental Section

### Biosynthesis of Aligned BNC (A‐BNC) and BNC Films—Biosynthesis of A‐BNC and BNC films

*Komagataeibacter xylinus* (*K. xylinus*) strain colonies (NCIMB 5346 from CECT, Valencia, Spain) were grown confluent on Hestrin–Schramm (HS) solid media^[^ ^13^ ^]^ in Petri dishes. The HS solid media consists of 1.15 g L^−1^ of citric acid, 6.80 g L^−1^ of Na_2_HPO_4_·12H2O, 5.00 g L^−1^ of peptone, 5.00 g L^−1^ of yeast extract, 15.00 g L^−1^ of agar, and 20.00 g L^−1^ of dextrose all from Condalab, dissolved and autoclaved in 1 L Milli‐Q water. HS liquid media contained the same components as the HS solid media but without agar (jelling agent). *K. xylinus* strain was cultured in HS solid media Petri plates for 15 days at 30 °C. Next, agar squares of ≈70 mm side with *K. xylinus* grown colonies were cut and placed carefully facing up with a sterile spatula at the bottom of the culture tube, previously filled with 10 mL sterile bacteria‐free liquid HS media with an oxygen‐permeable cap. Due to the oxygen availability that an aerobic bacteria strain (*K. xylinus*) require to survive, the dissolved oxygen (DO) concentration gradient formed in the culture tube is the main driving force of this method. The bacteria move toward the liquid–air interface to reach higher DO concentrations, leaving aligned BNC (A‐BNC) fibers behind. Hence, A‐BNC is synthesized with directionality toward the liquid–air interface of the culture. Once A‐BNC synthesis starts, and due to the bacteria “climbing up” the aligned BNC fibers, when the bacteria reach the liquid–air interface the typical hydrogel BNC pellicle. The system was cultured for 5 days at 30 °C under static conditions until a layer of BNC hydrogel (random fiber organization) was detected at the liquid–air interface. A‐BNC films were obtained within the length of the tube.

### Reuse of the Bacterial Substrates (Agar)

The confluence of bacterial colonies of the agar square used as bacterial substrate does not decrease. Therefore, the reuse of this piece of agar, which was carefully released from the produced cellulosic film and confined again at the bottom of a new culture tube filled with fresh liquid culture media (free of bacteria) (Figure 2G) was evaluated. The system was cultured for 3 days at 30 °C under static conditions until a layer of BNC hydrogel (random fiber organization) was detected at the liquid–air interface. A‐BNC films were obtained within the length of the tube. Agar substrates were reused for at least 7 cycles, obtaining A‐BNC and BNC films in only 3 days.

### “Film‐to‐Film” Biosynthesis; Reuse of BNC Film

The uncleaned BNC film produced at the liquid–air interface was detached from A‐BNC by pulling off the film with Teflon tweezers and cutting with sterilized scissors. Then, it was placed at the bottom of a new culture tube previously filled with fresh liquid culture media (free of bacteria). The system was cultured for 3 days at 30 °C under static conditions until a layer of BNC hydrogel (random fiber organization) was detected at the liquid–air interface. This results in films containing 2 circular BNC pellicles bound at the extremes of an A‐BNC film (a “Film‐to‐film,” Figure 2J). A‐BNC films were isolated. This process could be used repeated for at least 7 cycles, obtaining “Film‐to‐film's” consistently.

### Bacterial Nanocellulose Cleaning

Once produced, A‐BNC and BNC films were separated by carefully cutting and pulling out from the extremes of the films using Teflon tweezers. After separating the films, the cleaning process took place separately. BNC films were soaked and stirred with a magnetic stirring plate in the following steps^[^ ^46^, ^50^ ^]^: i) 1:1 Ethanol: Milli‐Q water solution for 10 min, ii) 40 min in boiling Milli‐Q water, and iii) two periods of 20 min in 0.1 m NaOH (Sigma‐Aldrich) aqueous solution. Finally, the BNC films were rinsed with Milli‐Q water until reaching neutral pH, autoclaved at 121 °C for 20 min and stored suspended in water in glass vials at room temperature. To avoid entanglement of the aligned fibers, A‐BNC films were gently cleaned with the same steps as BNC but without magnetic stirring.

### Bacterial Nanocellulose Drying

BNC samples were placed between two Teflon plates on a horizontal surface and dried at 60 °C for at least 12 h under a 1 kg weight. The thickness of the BNC films of 300 µm in wet decreased to ≈20 µm upon drying. As A‐BNC films are thinner than BNC, and thus more difficult to detach from a surface. Thus, A‐BNC films were dried on top of a Teflon plate on a horizontal surface and without any applied weight at 60 °C for at least 12 h. Neither drying with nor without weight modified the alignment of the A‐BNC films (data not shown).

### Biosynthesis of Aligned BNC (A‐BNC) and BNC Films—Atomic Force Microscopy (AFM)

A‐BNC and BNC films were dried on top of a thin conductive Si surface. 10 × 10 µm^2^ micrographs were obtained using a modular PM/AFM (Keysight 5500 LS SPM/AFM), using tapping mode. The isotropy distribution of AFM images was computed with MountainView8 (Digital Surf).

### Contact Angle (CA)

3 mm width and 10±2 µm thick dry A‐BNC and BNC films were fixed flat on a Teflon plate with tape. The surface wettability and capillarity of the materials were assessed by a contact angle measurer (KRÜSS Drop Shape Analyzer DSA 100), using the sessile drop method. 2 µL of Milli‐Q water were placed on the BNC surface and, after the water drop was deposited, CA values were computed over 0.5 for 10 s.

### Dissolved Oxygen (DO) Content

The reagent Leuco‐indigo carmine was used to quantify the dissolved oxygen (DO) content in liquid HS culture media. The Leuco‐indigo carmine method is a simple, rapid, and accurate colorimetric procedure for determining small amounts of DO in a liquid sample (0–50 ppm).^[^ ^48^, ^49^ ^]^ First, a solution of 3.6 mg mL^−1^ of Milli‐Q water containing the powder reagent (Indigo‐5,5’‐disulfonic acid disodium salt, Sigma‐Aldrich) and 0.04 mg mL^−1^ of dextrose (Condalab) was prepared (final volume of 2.5 mL). At the same time, 10 mL of 37.5% of KOH in water (Sigma‐Aldrich) were mixed with 37.5 mL of ethylene glycol (Sigma‐Aldrich). Both mixtures were added together and refrigerated until the Indigo‐Carmine reagent was reduced entirely. This could be noted due to the color change: starting from blue to red, and finally turning yellow (Figure S3A, Supporting Information). Test samples were collected with a syringe from the bottom and the top of the culture media from a 30 cm long culture vessel at days 1 and 7 of culture. 0.1 mL of the reduced solution of Indigo‐Carmine was added to a 7.5 mL test sample inside a vacuum container. The presence of DO oxidized the reagent and made the solution change color. The variation in color of the dye is directly proportional to the amount of DO present in the sample, which can quantitatively be determined by using a color scale (Figure S3B, Supporting Information). The DO concentration at different time points (days 1 and day 7) of bacterial culture and different culture locations (top and bottom) was evaluated. The volume extracted was evaluated with the Leuco‐indigo carmine reaction.

### Fourier‐Transformed Infrared (FTIR) Spectrophotometry

Dry A‐BNC and BNC films were analyzed with an FT/IR spectrophotometer (Jasco 4700LE). The transmittance spectra collection was performed at 2 cm^−1^. For each spectrum, 32 scans were coadded over the measuring range 400–4000 cm^−1^. Air was used as blank (machine lid open). The spectra were processed with the Spectra Manager Suite software to reduce CO_2_ and H_2_O noise levels, baseline correction, smoothing and peak find.

### Optical Microscopy

The morphology and structure of A‐BNC and BNC were assessed using a conventional optical microscope (Olympus RXSITRF 52787, MAB INDUSTRIAL). A retardation slide accessory made of a birefringent material (Olympus U‐PO3) was coupled to the optical microscope to obtain polarized microscopy images. For observing the change of light passing through the dry A‐BNC and BNC films, the polarized light direction was kept constant, and the sample was circularly rotated in the same plane within a turning center point for 360°, perpendicularly to the incident polarized light beam. Photos were taken every 45°.

### Fiber Orientation Analysis

The A‐BNC fibers orientation was analyzed using the *OrientationJ* Java plugin^[^ ^74^ ^]^ for ImageJ/FIJI (D. Sage, EPFL, 2.0.5 version). The *OrientationJ* structure tensor computes the orientation and isotropy properties in a local window. The local window is characterized by a 2D Gaussian function of standard deviation *σ*, based on the evaluation of the structure tensor in a local neighborhood. The parameter *σ* (expressed in pixel units) is a critical parameter that determines the scale of the analysis. It should have a value roughly close to the structure of interest (e.g., thickness of the cellulose filaments). The smallest local window available was 1 px, which was the set value used for this analysis. From the plugin, the “Color Analysis,” “Distribution,” and “Dominant Direction” modes were used to analyze images. *OrientationJ* was also used to obtain maximum frequencies from the angle distribution to assess the orientation coherency in several imaging techniques (Equation (1)).

(1) $$\mathrm{Orientation}\;\mathrm{coherency}\;=\;2\langle \cos^{2}\alpha \rangle \;-1$$

where *α* represents the angle between an individual fiber and the average fiber orientation. The coherency ranges from 0 to 1, where 0 represents an isotropic image without any preferential orientation and 1 represents a perfectly aligned distribution image.^[^ ^31^ ^]^

### Polarization‐Resolved Second‐Harmonic Generation Microscopy (PSHG)

Semiwet A‐BNC and BNC films were stained for 5 min with 5 µL with an aqueous solution of 0.25 mg mL^−1^ *Brightener 28* (seen as bright blue, exc 365 nm) in Milli‐Q water. Then, they were observed under a custom‐made multiphoton microscope system, which works both as a two‐photon excitation fluorescence microscope (TPEF) and as a polarization‐resolved second‐harmonic generation microscope (PSHG) (SLN Research Facility, Dr. Pablo Loza‐Alvarez, Institute of Photonic Sciences, Castelldefels, Spain).^[^ ^53^ ^]^ The illumination source is a Ti Sapphire oscillator that generates a linearly polarized laser of a wavelength of 810 nm in a frequency of 76 MHz and a 180 fs pulse. The laser then gets reflected in Galvo mirrors and the rotation of such linearly polarized light is performed by the rotation of a *λ∕*2 plate, to illuminate the sample by different angles of excitation, from 0° to 180°. The linear polarization of the incident beam was evaluated from 0° to 180° in steps of 10° using a lambda/2 plate, taking frames at each step. After reflection in a dichroic mirror, the beam excites the sample through an inverted microscope objective (28.25X Olympus). Immersion oil was added. By reflection, the TPEF signal is obtained backward after reflecting Nikon fluorescence cubes and passing through an IR filter. SHG signal is captured by a microscope objective (NA = 1.1) and acquired after reflecting a dichroic mirror that rejects backscattered laser light and modifies the ellipticity of the polarization states and passing through narrowband and IR filters. Brightfield, TPEF, and PSHG image stacks were obtained from 0° to 180°, as well as Z stacks. Further analysis was performed pixel by pixel using the stack options “Z project” and “plot Z‐axis profile” from the heat map filtered image stacks in ImageJ. The *OrientationJ* ImageJ plugin^[^ ^74^ ^]^ was used to assess the orientation coherency of the fibers in the images (Equation (1)).

### Interferometry

A‐BNC and BNC films were dried on top of a glass coverslip. 3D images of 450 × 350 × 1.50 µm^3^ (A‐BNC) and 85 × 100 × 1.50 µm^3^ (BNC) were obtained with a high‐resolution dual‐core confocal and interferometry profilometer (Leica DCM 3D optical profilometer), using a “retouching surface” operator.

### Scanning Electron Microscopy (SEM)

Images were obtained using a high‐performance SEM (QUANTA FEI 200 FEG‐ESEM) without metallization at EHTs of 5–10 kV and 50 Pa (low vacuum conditions). A‐BNC and BNC films were fixed flat on aluminum SEM holders with adhesive carbon tape.

### Small Angle X‐Ray Scattering (SAXS)

SAXS is commonly used to evaluate nanofibers’ anisotropy precisely.^[^ ^34^, ^38^ ^]^ The scattering measurements were performed at the NCD‐SWEET beamline at ALBA Synchrotron light facility in Cerdanyola del Vallès (Barcelona). A monochromatic X‐ray beam of 8 keV (*λ* = 0.154 nm) was set using a Si (1 1 1) channel‐cut monochromator. An array of Be lenses was employed to collimate the X‐ray beam, obtaining a beam size at the sample position of 50 × 150 µm^2^ (V × H). The scattering patterns were recorded using a Pilatus3 S 1M detector (Dectris), which consists of a pixel array of 1043 × 981 (V × H) with a pixel size of 172 × 172 µm^2^. The scattering vector *q* (defined as *q* = 4*π*sin(*θ*)/*λ*, being *θ* is the scattering angle and *λ* is the X‐rays wavelength)^[^ ^75^ ^]^ was calibrated using Silver Behenate as reference, obtaining a sample to detector distance of 6700 mm. Dry A‐BNC and BNC samples were fixed flat on 0.50 cm Ø metal rings by the edges. Samples were scanned perpendicularly to the beam direction, with an acquisition time of 30 s. Azimuthal integration of the obtained signal was done using PyFAI,^[^ ^76^ ^]^ limited by 270° of azimuthal range due to the intrinsic detector gaps limitations but enough to evaluate the 180° of the twofold fiber symmetry. The azimuthal distribution against the maximum value was normalized. The integrated scattering pattern generated a distribution of the diffraction intensity along the Debye‐Scherrer ring, I(Φ) (arb. u.), versus azimuthal angle, Φ (°). A fitting of the distributions was performed using the OriginLab (OriginLab Corporation) nonlinear function Lorentz equation (Equation (2)) from where the orientation parameters FWHM, OI, and S (Equations (3)–(5)) could be obtained to quantify the alignment of the BNC nanofibers.^[^ ^34^, ^38^ ^]^

FWHM stands for the full width of the half‐maximum intensity of the azimuthal profiles from the selected diffraction and it is obtained directly from the fitting. FWHM ranges from 0° to 180° and low values are correlated with the higher orientation of the sample.^[^ ^34^, ^38^ ^]^ OI (also as *f*_c_) is defined as an orientation index and complementary to FWHM. The OI ranges from 0 to 1, with 0 describing a random arrangement and 1 describing a perfect nanofibrils orientation. S is Herman's order parameter, obtained with MatLab using Equations (4)–(6). Similar to OI, a value of S = 0 means the nanofibers are randomly oriented, while *S* = 1 indicates a full nanofiber alignment.

(2) $$y\;=\;y_{0}\;+\;\frac{2\;A}{\pi }\;\cdot \;\frac{\omega }{4{\left(x-x_{c}\right)}^{2}+\omega^{2}}$$

(3) $$OI\;=\;\frac{180^{\circ }\;-\;\mathrm{fwhm}}{180^{\circ }}$$

(4) $$S\;=\;\frac{3\;\cos^{2}\Phi_{c,z}\;-\;1}{2}$$

(5) $$\cos^{2}\Phi_{c,z}\;=\;1\;-\;2\;\cos^{2}\Phi_{200,z}$$

(6) $$\cos^{2}\Phi_{200,z}\;=\;\frac{\sum_{0}^{\pi }I\left(\Phi \right)\;\sin\Phi \;\cos^{2}\Phi }{\sum_{0}^{\pi }I\left(\Phi \right)\;\sin\Phi }$$

### Thermal Conductivity

The thermal conductivity, k, of a material is a measure of its ability to conduct heat.

Thermal conductivity measurements of A‐BNC and BNC were performed using the 3*ω*‐method and a novel contactless frequency‐domain approach designed Pérez et al.,^[^ ^66^ ^]^ and labeled after Anisotropic Thermoreflectance Thermometry (ATT). The latter methodology is particularly suitable to study thermally anisotropic materials (e.g., aligned BNC) since it delivers the angular distribution of the thermal conductivity perpendicular to the surface of the sample. Measurements were performed with the described system on A‐BNC films dried on silicon wafers at room temperature, to which a gold coating was added.

The 3*ω*‐method is based on electrically heating a thin planar resistor using an AC harmonic current I0 at a frequency *ω*, and subsequently measuring the resultant voltage drop at the first (V*ω*) and third (V3*ω*) harmonics. By defining the normalized temperature coefficient of resistance as Equation (7) with R0 the resistance of the resistor at the temperature T0, the amplitude of the AC component of the temperature oscillations induced can be determined as Equation (8).

(7) $$\beta \;\equiv \;\left(1/R0\right)\left[\partial R/\partial T\right]$$

(8) $$\Delta TAC\;=\;2\left|V3\;\omega \left|\beta \right|V\;\omega \;\right|$$

By solving the 2D heat equation for the geometry of a linear heat source supported on a semi‐infinite medium, the thermal conductivity *κ* of such medium can be obtained as Equation (9).

(9) $$\kappa =-P02\pi l1\left(\partial \Delta \mathrm{TAC}/\partial \ln2\omega \right)$$

With P0 the total dissipated power at the resistor and l the length of the resistor. Since the AC frequency determines the thermal penetration depth according to 1/q=√α/i2ω, for thick films supported on a semi‐infinite medium (i.e., a substrate) the ΔTAC versus ln 2*ω* curve shows low and mid‐frequency regimes that primarily correspond to the substrate and the supported film, respectively. Obtained from the slope (∂ΔTAC/∂ln2ω) of both regions, this method is straightforward to determine the thermal conductivity.

Statistics were performed on 7 different resistors thermally evaporated on BNC films yielded to obtain the thermal conductivity.

Transmission electron microscopy (TEM). A‐BNC and BNC films were dried on top of a copper TEM grid. Images were obtained using a high angular range TEM (JEOL 1210 TEM), operating at 120 kV with an ORIUS 831 SC 600 Gatan camera. SAED was used to obtain the diffraction pattern from the A‐BNC and BNC films, to assess its crystallinity.

Evaluation of A‐BNC and BNC as Cell Carriers: in vitro experiment. A human dermal fibroblast (hDF) cell line (1BR.3.G, ECACC 90020507) was used to evaluate the performance of A‐BNC and BNC as adherent cell carriers, and how the structuration of A‐BNC influences the cell growth. Cells were maintained in Dulbecco's Modified Eagle Medium (DMEM; Gibco) supplemented with 2 mm GlutaMax (Gibco), and 10% fetal bovine serum (FBS; Gibco) at 37 °C in 10% CO_2_. The subculture routine for 1BR.3.G consisted of split sub‐confluent cultures (70–80%) 1:3–1:6, i.e., seeding at 2–4×10 000 cells cm^−^^2^ using 0.05% trypsin/EDTA; 10% CO2; 37 °C. hDF were cultured on dry sterile samples of A‐BNC and BNC films. In brief, cellulose samples were autoclaved (121 °C, 20 min) and dried on autoclaved glass microscope slides at room temperature in a laboratory hood for 6 h, and finally irradiated with UV light for 30 min to ensure sterility. Slides were placed in Ø 8.5 cm Petri dishes and preconditioned with cell media, before seeding 1.5 m cells per dish. Cell media (20 mL) was changed every 2 days. After 4 and 7 days of culture, images were obtained with an inverted microscope (Nikon Eclipse Ts2). From optical microscope images, the orientation coherency was measured with the *OrientationJ* ^[^ ^74^ ^]^ plugin for Image*J* (Equation (1)).

### Statistical Analysis

Quantitative data are expressed as means ± standard deviation. Statistical analyzes were performed with Graph Pad Prism 8 software using one‐way ANOVA followed by Tukey's multiple comparison test. Statistical significance was accepted when *P*‐values were ≤0.05 and summarized as * = *P* ≤ 0.05, ** = *P* ≤ 0.01, *** = *P* ≤ 0.001, **** = *P* ≤ 0.0001 for the calculated *P*‐values.

## Conflict of Interest

The authors declare no conflict of interest.

## Author Contributions

N.M.: Methodology, Validation, Formal analysis, Investigation, Visualization, Writing – original draft, review & editing. S.R.‐S.: Methodology, Writing – editing. I.A.‐S.: Methodology, Writing – editing. N.M.: Investigation, Visualization, Writing: editing. K.X.: Investigation, Visualization, Writing: editing. E.S.: Resources, Investigation, Visualization, Writing: review & editing. J.S.R.: Resources, Investigation, Visualization, Writing: review & editing. A.L.: Conceptualization, Funding acquisition, Supervision, Methodology, Validation, Formal analysis, Investigation, Visualization, Writing – original draft, review & editing.

## Supporting information

Supporting Information

## Data Availability

The data that support the findings of this study are available from the corresponding author upon reasonable request.
