# Supplementary material for: Highly Aligned Bacterial Nanocellulose Films Obtained During Static Biosynthesis in a Reproducible and Straightforward Approach
Source: Adv Sci (Weinh). 2022 Jul 21;9(26):2201947. doi: 10.1002/advs.202201947 (PMC9475533; doi:10.1002/advs.202201947)
Supplement: Supplementary file 1 — Supporting Information [file ADVS-9-2201947-s001.pdf]

ADVANCED SCIENCE
Open Access

Supporting Information

for *Adv. Sci.*, DOI 10.1002/advs.202201947

Highly Aligned Bacterial Nanocellulose Films Obtained During Static Biosynthesis in a Reproducible and Straightforward Approach

*Nerea Murugarren, Soledad Roig-Sanchez, Irene Antón-Sales, Nanthilde Malandain, Kai Xu, Eduardo Solano, Juan Sebastian Reparaz and Anna Laromaine**

Supporting information:

Highly aligned bacterial nanocellulose films obtained during static biosynthesis in a reproducible and straightforward approach

Nerea Murugarren[a], Soledad Roig-Sanchez[a], Irene Antón-Sales[a], Nanthilde Malandain[a], Kai Xu[a], Eduardo Solano[b], Juan Sebastian Reparaz[a], Anna Laromaine[a]

a. Institut Ciencia de Materials de Barcelona (ICMAB-CSIC), Campus UAB, 08193 Bellaterra, Spain.

b. NCD-SWEET beamline, ALBA Synchrotron Light Source, Carrer de la Llum 2−26, 08290 Cerdanyola del Vallès, Barcelona, Spain.

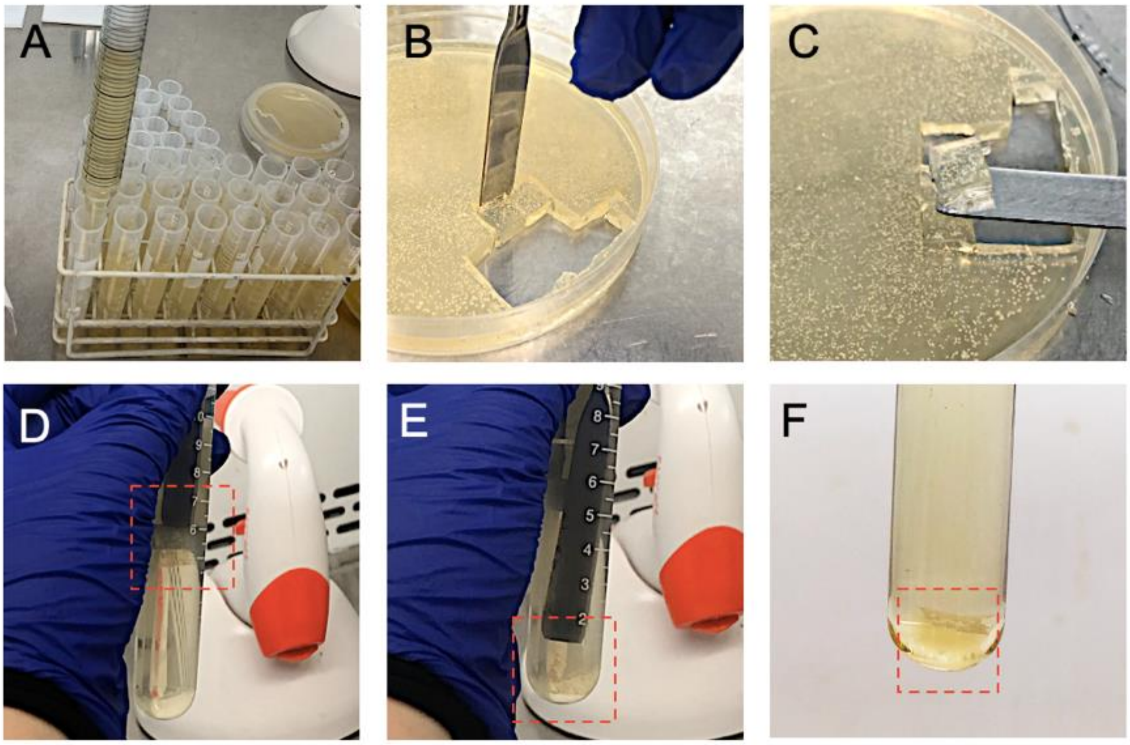

Figure S1. Laboratory procedure to obtain aligned BNC (A-BNC). A Culture tubes are filled with culture media. **B-C** Agar squares containing bacteria are cut and collected with a sterile spatula. **D-E** The agar square is placed carefully inside the culture tube containing culture media and carefully pushed to the bottom of the tube with the spatula. **F** Agar squares are finally displayed with the bacterial colonies facing up during A-BNC culture. Bacterial colonies do not detach from the agar squares during the whole procedure. Agar squares with bacteria indicated in red discontinuous squares.

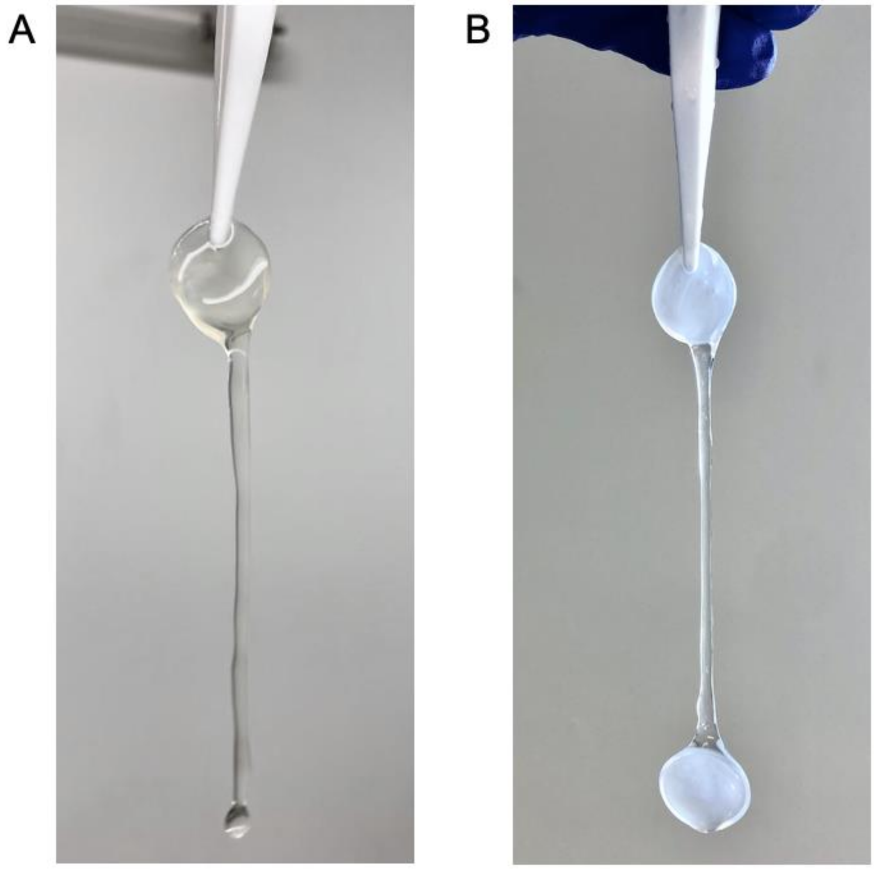

Figure S2. Aspect of wet A-BNC and "Film-to-film" samples. Image of cleaned and wet **A** A-BNC and **B** "Film-to-film" samples held by Teflon tweezers. The samples are freestanding; they present stable structure and composition when held from an extreme. Color differences may vary depending on the room´s light conditions.

A

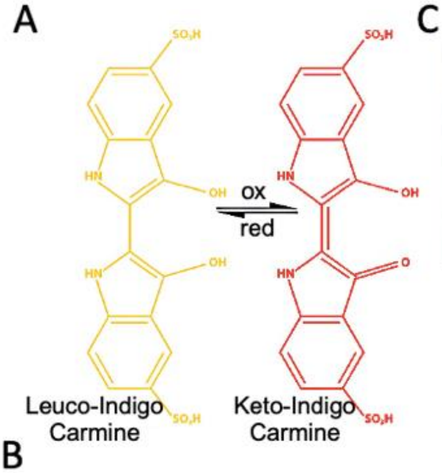

B

Description of color	$[O_2]_{diss}$ (p.p.m)
Strong yellow (red.)	0.00
Orange	0.03
Red	0.06
Purple	0.09
Blue	0.12
Strong blue-green (ox.)	0.15

C

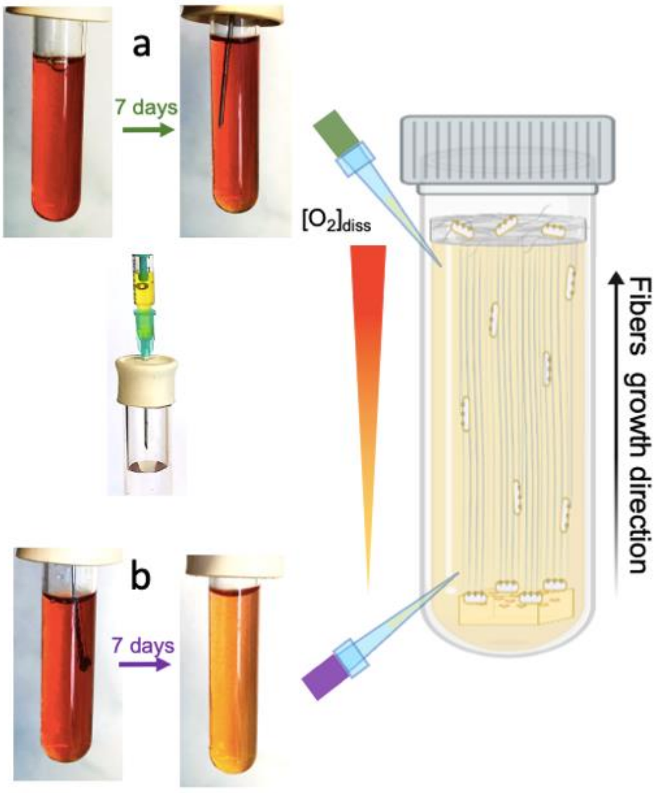

Figure S3. Dissolved Oxygen (DO) colorimetry assay. A Leuco- indigo carmine reagent oxidizes under the presence of oxygen to keto-indigo carmine, which causes a color change from yellow to red upon oxidation. **B** A color-DO chart allows to quantitatively estimate the DO concentration. **C** We evaluated the DO concentration at different time points (day 1 and day 7) of bacterial culture and at different locations of the culture (top and bottom). We took a sample from the bacterial culture on day 1 from top and bottom using a long syringe. The volume extracted was evaluated with the leuco-indigo carmine reaction. We repeated this process after 7 days of culture. **a** shows the leuco-indigo carmine color obtained of the top part of the bacterial culture on day 1 and day 7. The color obtain indicated a DO concentration of ≈0.06 ppm, seen as a red solution and which did not change upon time. **b** shows the leuco-indigo carmine color obtained of the bottom part of the bacterial culture on day 1 and day 7. The color obtained indicated that DO concentration decreased to 0.03 ppm from the initial DO concentration of ≈0.06 ppm.

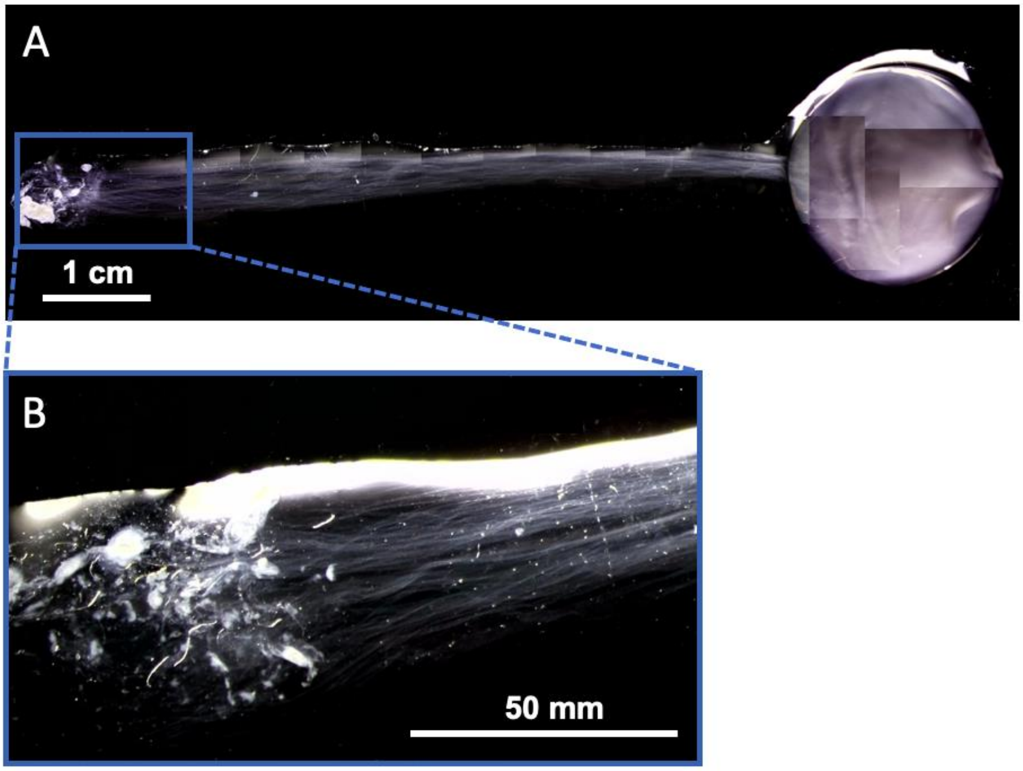

Figure S4. Residual bacterial colonies in an A-BNC and BNC film. A,B Optical microscope images of an uncleaned wet A-BNC and BNC film where bacterial colonies can be observed at the extreme. The film was pulled from the agar, where the bacterial colonies were anchored.

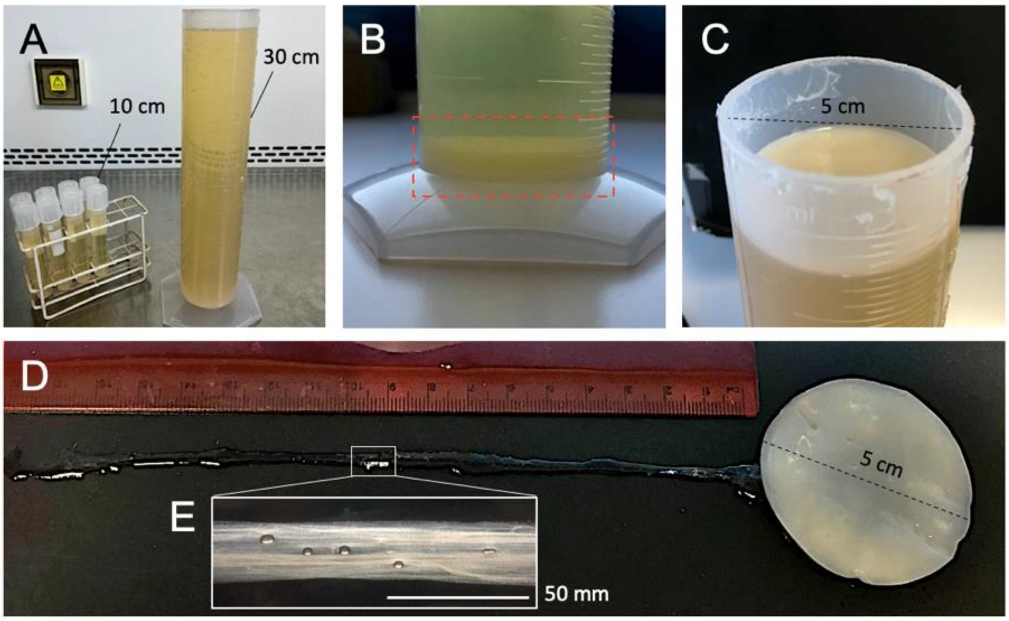

Figure S5. Methodology to obtain larger A-BNC films. A Size comparison from small (10 cm long) vessels to large (30 cm long) vessels used in this study. **B** Bottom of the large vessel, containing an agar piece with bacterial colonies facing up. **C** BNC film produced in the liquid-air interface after 5 days of culture. **D** A-BNC and BNC film. The BNC films had a diameter of 5 cm and the A-BNC films obtained using large vessels had a length of 17-20 cm. **E** Optical microscope image from a section of D.

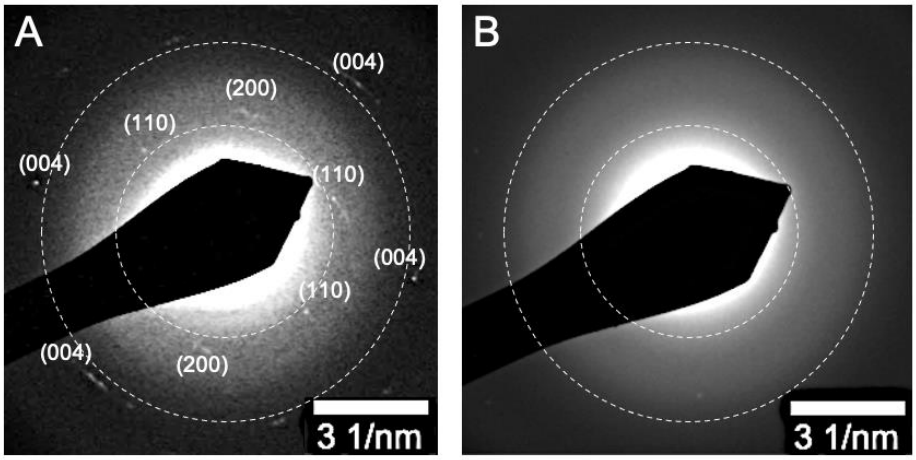

Figure S6. Selected Area Electron Diffraction (SAED) of A-BNC and BNC. Selected Area Electron Diffraction (SAED) of **A** A-BNC and **B** BNC. The cellulose crystalline lattice planes are only seen and indexed in **A**.

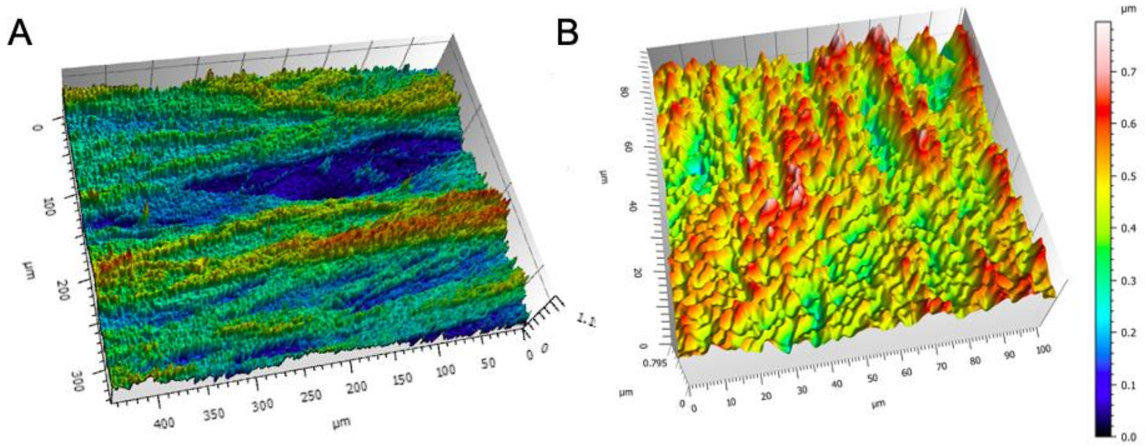

Figure S7. 3D Micro-structuration of BNC and A-BNC. Interferometry images of **A** A-BNC (350 x 450 μm^2) and **B** BNC (85 x 100 μm^2) sample sections. Color map showing the height of the section**.**

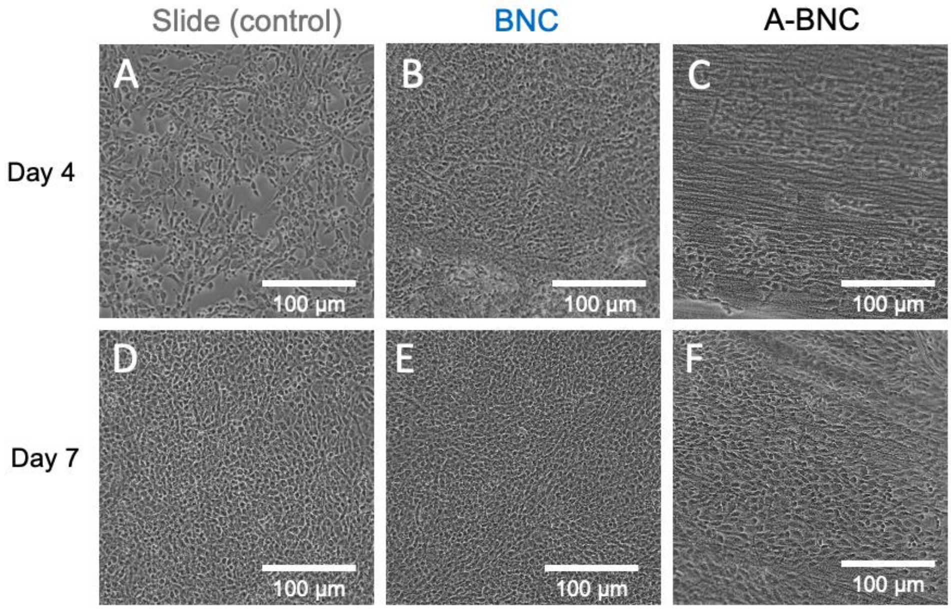

Figure S8. Optical images of cell culture for slide (control), BNC and A-BNC. Top row shows day 4 images of **A** slide, **B** BNC and **C** A-BNC. Bottom row shows day 7 images of **D** slide, **E** BNC and **F** A-BNC.
